# Supplementary material for: Evaluation and mitigation of cognitive biases in medical language models
Source: NPJ Digit Med. 2024 Oct 21;7:295. doi: 10.1038/s41746-024-01283-6 (PMC11494053; doi:10.1038/s41746-024-01283-6)
Supplement: Supplementary file 1 — Supplemental information and tables [file 41746_2024_1283_MOESM1_ESM.pdf]

Supplementary Information for *Evaluation and mitigation of cognitive biases in medical language models*

Model	No bias	Bias							Avg.
		Self-diagnosis	Recency	Confirmation	Frequency	Cultural	Status quo	False consensus	
`gpt-4-0613`	0.727	0.698	0.679	0.725	0.627	0.681	0.679	0.640	0.676
`mixtral-8x7b-instruct-v0.1`	0.518	0.460	0.455	0.504	0.373	0.421	0.445	0.428	0.441
`gpt-3.5-turbo-0613`	0.497	0.288	0.333	0.458	0.274	0.277	0.361	0.239	0.319
`PaLM-2`	0.429	0.322	0.232	0.358	0.167	0.231	0.220	0.223	0.250
`llama-2-70B-chat`	0.357	0.169	0.141	0.219	0.104	0.207	0.160	0.090	0.156
`pmc-llama-13b`	0.334	0.197	0.247	0.218	0.224	0.278	0.290	0.214	0.238

Supplementary Table 1: No bias mitigation. Tabular results for the performance (i.e., accuracy) of each LLM model without bias mitigation. The "no bias" column indicates that no bias was injected into the question, but all else was the same. The remaining columns indicate each of the seven cognitive biases we considered.

Model	Bias								
	No bias	Self-diagnosis	Recency	Confirmation	Frequency	Cultural	Status quo	False consensus	Avg.
gpt-4-0613	0.727	0.728	0.709	0.744	0.720	0.681	0.725	0.709	0.717
mixtral-8x7b-instruct-v0.1	0.518	0.503	0.513	0.497	0.477	0.391	0.529	0.477	0.484
gpt-3.5-turbo-0613	0.497	0.448	0.391	0.493	0.370	0.274	0.430	0.252	0.380
PaLM-2	0.429	0.435	0.271	0.382	0.237	0.261	0.239	0.317	0.306
llama-2-70B-chat	0.357	0.319	0.204	0.230	0.185	0.213	0.286	0.113	0.221
pmc-llama-13b	0.334	0.216	0.247	0.246	0.231	0.233	0.292	0.192	0.237

Supplementary Table 2: Bias mitigation using education strategy. Same as Supplementary Table 1, but for education mitigation.

Model	Bias								
	No bias	Self-diagnosis	Recency	Confirmation	Frequency	Cultural	Status quo	False consensus	Avg.
gpt-4-0613	0.763	0.738	0.742	0.734	0.720	0.698	0.741	0.744	0.731
mixtral-8x7b-instruct-v0.1	0.513	0.466	0.417	0.487	0.353	0.380	0.418	0.437	0.423
gpt-3.5-turbo-0613	0.505	0.316	0.355	0.437	0.350	0.258	0.381	0.437	0.362
PaLM-2	N/A	N/A	N/A	N/A	N/A	N/A	N/A	N/A	N/A
llama-2-70B-chat	0.325	0.191	0.179	0.232	0.098	0.165	0.163	0.160	0.170
pmc-llama-13b	0.317	0.185	0.221	0.312	0.165	0.210	0.227	0.217	0.220

Supplementary Table 3: Bias mitigation using one-shot mitigation strategy. Same as Supplementary Table 1, but for one-shot mitigation. For the one-shot and few-shot tables, we note that the safety filters prevented PaLM-2 from answering the vast majority of questions, so we exclude it from our analyses (see "Methods" for details).

Model	No bias	Bias							Avg.
		Self-diagnosis	Recency	Confirmation	Frequency	Cultural	Status quo	False consensus	
gpt-4-0613	0.757	0.749	0.764	0.762	0.749	0.726	0.770	0.753	0.752
mixtral-8x7b-instruct-v0.1	0.531	0.478	0.408	0.463	0.341	0.369	0.422	0.405	0.412
gpt-3.5-turbo-0613	0.507	0.391	0.342	0.486	0.414	0.273	0.412	0.277	0.371
PaLM-2	N/A	N/A	N/A	N/A	N/A	N/A	N/A	N/A	N/A
llama-2-70B-chat	0.308	0.141	0.130	0.143	0.072	0.137	0.134	0.047	0.115
pmc-llama-13b	0.334	0.213	0.220	0.272	0.206	0.210	0.257	0.246	0.232

Supplementary Table 4: Bias mitigation using few-shot mitigation strategy. Same as Supplementary Table 1, but for few-shot mitigation.

Supplementary Note 1

For each model, we solicited model responses in the form of a single letter, as described in Methods. However, some models refused to answer a subset of questions; the proportion of non-response rates are shown in Supplementary Table 5. For concision, we only show non-response for prompts *without* cognitive bias injection; non-response rates were similar for prompts with and without cognitive biases. For most analyses, we removed these non-response results from our analyses (i.e., reported accuracy was adjusted to exclude non-response answers). However, because the one- and few-shot non-response was very high for `PaLM-2` (0.944 and 0.995, respectively), we exclude these results from our analyses entirely.

We observed that non-response for `PaLM-2` was due to triggering safety filters, while for `llama-2-70B-chat` and `pmc-llama-13b` it was because the model provided nonsensical answers, multiple answers, or refused to answer the question entirely. In the case of `PaLM-2`, for example, we observed a randomly-selected few-shot example to `PaLM-2` was blocked because it fell under the safety category `HarmCategory.HARM_CATEGORY_MEDICAL` and was judged to have high harm probability (`HarmProbability.HIGH`). This effect was particularly pronounced for one- and few-shot mitigation. For `llam-2-70B-chat` and `pmc-llama-13b`, we relied on an auto-evaluation approach (see "Methods" for details) to extract selected choices from the model's output; in the case that no clear answer was given, the response was judged to be a non-response.

	Mitigation strategy		
Model	No mitigation	One-shot	Few-shot
gpt-4-0613	0.000	0.000	0.000
mixtral-8x7b-instruct-v0.1	0.001	0.001	0.001
gpt-3.5-turbo-0613	0.000	0.000	0.000
PaLM-2	0.266	0.944	0.995
llama-2-70B-chat	0.059	0.097	0.131
llama-2-70B-chat	0.187	0.219	0.302

Supplementary Table 5: Non-response rate by prompting strategy. Each cell shows the proportion of non-responses by mitigation strategy and model.
